# Developing Telemental Health Partnerships Between State Medical Schools and Federally Qualified Health Centers: Navigating the Regulatory Landscape and Policy Recommendations

**DOI:** 10.1111/jrh.12323

**Published:** 2018-10-04

**Authors:** John C. Fortney, Richard C. Veith, Amy M. Bauer, Paul N. Pfeiffer, Marcia Valenstein, Jeffrey M. Pyne, Gregory W. Dalack, Teresa L. Kramer, Lori J. Ferro, Karla Metzger, Jay H. Shore, Andrew D. Carlo, Sara Coates, Susan Ward‐Jones, Ed Larkins, Erin Hafer, Stephanie Shushan, Donald L. Weaver, Jürgen Unützer

**Affiliations:** ^1^ Department of Psychiatry and Behavioral Sciences, School of Medicine University of Washington Seattle Washington; ^2^ Department of Veterans Affairs, Health Services Research and Development Center of Innovation for Veteran‐Centered and Value‐Driven Care Seattle Washington; ^3^ Department of Psychiatry University of Michigan Medical School Ann Arbor Michigan; ^4^ VA Center for Clinical Management Research VA Ann Arbor Healthcare System Ann Arbor Michigan; ^5^ Department of Psychiatry, College of Medicine University of Arkansas for Medical Sciences Little Rock Arkansas; ^6^ Department of Veterans Affairs, Health Services Research and Development Center for Mental Healthcare and Outcomes Research Little Rock Arkansas; ^7^ Helen and Arthur E. Johnson Depression Center University of Colorado Anschutz Medical Campus Aurora Colorado; ^8^ Michigan Primary Care Association Lansing Michigan; ^9^ East Arkansas Family Health Center West Memphis Arkansas; ^10^ Family Medical Center of Michigan Temperance Michigan; ^11^ Community Health Plan of Washington Seattle Washington; ^12^ National Association of Community Health Centers Bethesda Maryland

**Keywords:** access, mental health, policy, safety net clinics, telehealth

## Abstract

**Background:**

Federally Qualified Health Centers (FQHCs) deliver care to 26 million Americans living in underserved areas, but few offer telemental health (TMH) services. The social missions of FQHCs and publicly funded state medical schools create a compelling argument for the development of TMH partnerships. In this paper, we share our experience and recommendations from launching TMH partnerships between 12 rural FQHCs and 3 state medical schools.

**Experience:**

There was consensus that medical school TMH providers should practice as part of the FQHC team to promote integration, enhance quality and safety, and ensure financial sustainability. For TMH providers to practice and bill as FQHC providers, the following issues must be addressed: (1) credentialing and privileging the TMH providers at the FQHC, (2) expanding FQHC Scope of Project to include telepsychiatry, (3) remote access to medical records, (4) insurance credentialing/paneling, billing, and supplemental payments, (5) contracting with the medical school, and (6) indemnity coverage for TMH.

**Recommendations:**

We make recommendations to both state medical schools and FQHCs about how to overcome existing barriers to TMH partnerships. We also make recommendations about changes to policy that would mitigate the impact of these barriers. Specifically, we make recommendations to the Centers for Medicare and Medicaid about insurance credentialing, facility fees, eligibility of TMH encounters for supplemental payments, and Medicare eligibility rules for TMH billing by FQHCs. We also make recommendations to the Health Resources and Services Administration about restrictions on adding telepsychiatry to the FQHCs’ Scope of Project and the eligibility of TMH providers for indemnity coverage under the Federal Tort Claims Act.

There is an inequitable geographic distribution of mental health specialists in the United States, resulting in substantial unmet need in rural counties^1^ and a significant rural‐urban disparity in the receipt of specialty mental health care.^2^ The Centers for Medicare and Medicaid Services (CMS) define telemedicine as “the provision of clinical services to patients by physicians and practitioners from a distance via electronic communications.”^3^ Telemental Health (TMH) encounters include the delivery of psychotherapy and pharmacotherapy services, as well as consultations to establish diagnoses and provide treatment recommendations. There is a preponderance of evidence that TMH is effective across a wide range of diagnoses and populations.^4^ Though TMH is particularly well‐suited to delivering care from a distance,^5^ adoption has been negligible in Medicare, Medicaid, and the private insurance sector.^6^, ^7^, ^8^

TMH can be delivered with a range of intensities, from curbside consultation to referral for ongoing care.^9^, ^10^ The 2 most effective models of TMH are collaborative care and referral care.^9^ The TMH Collaborative Care model involves off‐site mental health providers collaborating and consulting with the primary care team to manage patients without providing treatment directly. The TMH Referral model involves the off‐site mental health team taking over the care of the patient (eg, prescribing and/or delivering psychotherapy). To be successful, both models of TMH require considerable investment in establishing technological infrastructure, administrative arrangements, clinical workflow, and billing processes. Fortunately, many of the technological and regulatory obstacles to TMH have been reduced in recent years,^11^ paving the way for widespread adoption. Importantly, TMH services can now be provided through less‐expensive web‐based platforms that are compliant with the Health Insurance Portability and Accountability Act (HIPAA).^12^

There are excellent guidelines for managing TMH encounters such as informed consent, HIPAA compliance, risk management, and indemnity.^5^, ^13^, ^14^ However, there is less guidance about establishing a sustainable TMH program. While large integrated health care systems such as the Veterans Health Administration have been able to successfully deploy TMH at scale,^15^, ^16^ small independent rural primary care practices have lagged behind. Even when TMH programs are successfully launched with grant funding, they often fail to transition to financial sustainability.^17^ In this paper, we share our experience launching the *Study to Promote Innovation in Rural Integrated Telepsychiatry* (SPIRIT) trial, a large pragmatic trial (PCS‐1406‐19295) comparing 2 approaches to TMH in Federally Qualified Health Centers (FQHCs) serving rural areas of Arkansas, Michigan, and Washington state. Supported by the Health Services and Resources Administration (HRSA), FQHCs deliver primary care services in areas where geographic, economic, and/or cultural barriers limit access to care. FQHCs are a key component of America's health care safety net and are essential partners in efforts to address health disparities. Nationwide, there are nearly 1,400 grantees with over 10,000 clinic locations that provide services to 26 million Americans.^18^ Almost half (44%) of FQHC patients live in rural areas,^19^ 92% live at or below 200% of the Federal Poverty Level,^20^ and 62% are racial/ethnic minorities.^20^ Few FQHCs offer telemedicine services.^21^ In the SPIRIT trial, TMH services were delivered to FQHCs by the departments of psychiatry at publicly funded state medical schools.

There is a growing movement to improve the social mission of medical schools to increase access to care for disadvantaged populations.^22^, ^23^ The mission statements of the medical schools involved in the SPIRIT trial clearly communicate the goal of serving all the residents of their respective states. TMH supports the core clinical, educational, and research missions of publicly funded state medical schools including: (1) *Clinical*—reaching all state residents, including those living in underserved communities; (2) *Education*—exposing trainees to complex disorders and disadvantaged populations^24^ and providing training in TMH delivery^25^; and (3) *Research*—including study participants from diverse backgrounds. Because FQHCs serve diverse populations with complex chronic illnesses, establishing TMH partnerships with state medical schools is mutually beneficial to both parties. FQHCs could also explore partnering with other health care organizations (eg, private medical schools, for‐profit TMH companies), though the missions may not overlap as well as with their state medical school.

It is critical that patients have the opportunity to receive TMH services without having to navigate to another health care system. Based on comparisons of telepsychotherapy use from 2 randomized controlled trials, navigation from one health care system to another appears to be a major barrier for patients. In a TMH trial conducted in the Veterans Health Administration (integrated care system), 54.9% of study participants had telepsychotherapy encounters.^26^ In a similar trial where FQHC patients had to enroll at the medical school to receive TMH services, only 16.6% of study participants had a telepsychotherapy encounter.^27^ Navigating to a different health care system entails logistical barriers (eg, intake paperwork burden, unfamiliar appointment scheduling system), as well as attitudinal barriers (eg, lack of trust, stigma), and privacy concerns (eg, medical records stored in multiple health care systems), all of which contribute to decreased patient engagement in TMH. Therefore, it is critical that FQHC patients have an opportunity to receive TMH services without having to navigate to another health care system.

The solution to this problem is to have the off‐site TMH providers practice as part of the FQHC team. This also substantially increases quality and safety because the TMH providers and the primary care team share the same Electronic Health Record (EHR). However, this solution creates other logistical and administrative problems, which have to be overcome. Specifically, it requires that the TMH providers become credentialed and privileged to practice at the FQHC, have access to and be trained on the FQHC's EHR, and be able to bill as an FQHC provider. In addition, while the TMH provider is covered by the medical school's indemnity plan, the FQHC as an entity has to secure malpractice coverage for TMH. Based on our experience launching the SPIRIT trial, the remainder of this paper describes the major barriers and solutions to establishing a sustainable TMH program between FQHCs and state medical schools that does not involve patients having to navigate to a different health care system. The following issues are addressed: (1) credentialing and privileging, (2) FQHC Scope of Project, (3) EHR remote access, (4) insurance credentialing/paneling, billing and supplemental payments, (5) contracting, and (6) indemnity. The paper concludes with a number of recommendations for policy changes that will help mitigate most of these barriers.

## Major Barriers and Solutions to Establishing a Sustainable TMH Program

### Credentialing and Privileging

HRSA defines credentialing as “the process of assessing and confirming the license or certification, education, training, and other qualifications of a licensed or certified health care practitioner.”^28^ HRSA defines privileging as “the process of authorizing a health care practitioner's specific scope and content of patient care services.”^28^ Credentialing and privileging TMH providers to practice at each FQHC is an expensive, burdensome, and time‐consuming (eg, 90‐120 days) process. Fortunately, in 2011 CMS and The Joint Commission both approved credentialing and privileging “by proxy” standards, which greatly streamlines the process.^29^ This proxy process allows the “originating‐site” receiving the telemedicine services to accept the “distant‐site's” credentialing and privileging decisions. However, FQHCs must amend their bylaws and be willing to accept the indemnity risk associated with the distant‐site's credentialing and privileging decisions.^3^ The FQHC must also ensure through a written agreement that specific requirements are met including that the FQHC reviews the TMH provider's performance and sends the distant site such performance information for use in their provider appraisals.^30^ The written agreements described above are complex, although templates are available.^31^ As a result, FQHCs typically use existing credentialing and privileging processes rather than amending bylaws, developing written agreements, and sharing information about providers.

### Scope of Project

HRSA requires that each FQHC have an approved Scope of Project, which specifies its sites, services, service area, and target population. Mental health services (including psychiatry) are appropriate for inclusion in an FQHC's Scope of Project.^32^ FQHCs must submit a Change in Scope application at least 60 days before adding TMH services and must implement the service within 120 days of approval.^33^ For HRSA to approve adding a new service, the FQHC must demonstrate how it will meet the health needs of the population served. A new clinical service can either be: (1) directly provided by the FQHC, (2) provided under a formal written contract, (3) provided by formal written referral arrangement, or (4) provided by an informal referral arrangement.^28^ In the first 2 scenarios, the FQHC can bill for the new service.

There are 3 important stipulations required for approval of Scope of Project changes, including the addition of TMH services. First, adding the new service must not require additional funding under the Section 330 Public Health Service Act Health Center Program grant.^34^ Thus, the FQHC must demonstrate that there will be adequate revenue to cover the added expense, and that it will be able to continue to maintain the level and quality of the required primary care services currently being provided.^32^ Second, the FQHC must describe how all current patients will have access to the new service. In the case of TMH, this may be difficult if all clinic locations do not have the necessary space and equipment or if there are large numbers of uninsured patients.^34^ Third, if the new service is to be provided via formal written contract and the FQHC plans to bill for the encounters, the application must specify how the encounters will be documented in the FQHC's EHR, and how the FQHC will bill for the service.^34^ Thus, to add TMH as a new service via formal written contract, the TMH providers at the state medical school must have remote access to the EHR and the TMH encounter must be billable to Medicaid (the primary insurer of FQHC patients).

### EHR Remote Access

For safety and quality assurance purposes, it is critical that the TMH providers have access to the FQHC's EHR. Direct access to the EHR allows the TMH providers to see current medications, lab results, and diagnoses that could influence the treatment plan. For telepsychiatrists, access to the EHR also allows them to e‐prescribe medications and order lab tests. In addition, by charting in the FQHC's EHR, the primary care team has convenient access to the results of the TMH provider's clinical assessment and treatment plan. Current electronic health information exchange technologies are not sufficient to ensure this level of quality and safety.

The cost of remote EHR access depends on how many sites and providers use the software and/or on how many computers the software is installed. Often the cost of the user license will depend upon the class of provider (eg, prescriber vs nonprescriber, whether the provider generates a billable encounter). Under the commonly used subscription license format, recurring costs include an annual subscription fee, which can range up to $10,000 per provider. Because TMH providers are part‐time, the return on the investment of a user license may not be economical. Unless FQHCs can negotiate discounted rates for part‐time TMH providers, the high cost of TMH user licenses represents a major barrier to adoption.

EHRs also have steep learning curves and there are major differences across systems. This limits the number of EHRs a TMH provider has the cognitive capacity to use on a day‐to‐day basis to about 3 or 4. Thus, medical schools should have TMH providers devote a small portion of their time to delivering TMH services to a few FQHCs (ie, point‐to‐point dispersed model) rather than having a few full‐time providers delivering TMH services to large numbers of FQHCs (ie, hub and spoke model). Ideally, the FQHCs assigned to a TMH provider would all be operating the same EHR. In addition to clinical documentation, EHRs are also used to schedule encounters. Having a TMH provider practice in multiple FQHCs with different EHRs complicates the scheduling process. Therefore, it may be necessary to create a shadow scheduling system in which encounters are scheduled in the FQHC's EHR and in a centralized scheduling system that multiple FQHCs use to make appointments with the TMH provider.

### Insurance Credentialing/Paneling, Billing, and Supplemental Payments

#### Insurance Credentialing/Paneling

To bill, the TMH provider must be empaneled as an in‐network provider for each insurance company. Insurance credentialing (or paneling) involves verifying the provider's education, training, experience, and competency. Even if the TMH provider is already on the insurer's panel as part of their medical school practice, the process will have to be duplicated at each FQHC in order to bill. The review process can take 60‐120 days.

#### Billing

To add TMH to the Scope of Project, the FQHC must demonstrate that there will be adequate revenue to cover the added expense, and thus the FQHC must be able to bill for this service. There are 2 billing scenarios for TMH encounters. In the first scenario, the FQHC has a written agreement with the distant‐site to provide TMH services, but it does not financially compensate the distant‐site. In this scenario, the TMH provider bills for the encounter and does not need to be credentialed and privileged at the FQHC nor have access to their EHR. The FQHC can bill for a facility fee that compensates them for the coordination of the encounter. In addition to requiring the patient to navigate to another health care system and not sharing an EHR, the financial disadvantage of this scenario is that TMH encounters are not eligible to receive supplemental Medicaid payments that FQHCs are eligible for under the Prospective Payment System (PPS) described below. In the second scenario, the FQHC has a written agreement with the distant‐site to provide TMH services and financially compensates the distant‐site. The contracted rate would need to cover the TMH provider's salary and benefits, as well as any overhead. Typically, the FQHC would prepurchase a set number of TMH hours per month. In this second scenario, the FQHC bills for both the encounter and the facility fee. The financial advantage of this scenario is that the service is eligible for the higher reimbursement rates associated with PPS. Billing codes and modifiers for TMH are described in Table 1. For Medicare patients, FQHCs are not authorized to serve as a distant‐site, and they may not bill for the TMH encounter or include the TMH encounter on their PPS cost report.^35^, ^36^ Therefore, the first billing scenario must be used for Medicare patients.

**Table 1:** Telemental Health Billing Codes

Provider/Encounter Type	Current Procedural Terminology (CPT) Code	Comments
Psychiatrist: initial assessment	Initial diagnostic evaluation CPT code 90792 (Level 1)	Requires a medical assessment
Psychiatrist: follow‐up	Evaluation and Management CPT codes 99213–99215 (Level 1)	Code depends on the length/complexity of the encounter. Add‐on psychotherapy code can be used.
Psychologist: psychotherapy	90832 (30 minutes) (Level 1)	
	90833 (45 minutes) (Level 1)	
	90837 (60 minutes) (Level 1)	
Interactive video indicator	GT modifier (eg, 90792 GT)^a^	
Place of Service (POS) Code 02	Indicates that the encounter was conducted synchronously via interactive video	
Practice address	FQHC address	Required to be eligible for PPS supplemental payment
Originating site fee	Q3014 (Level II)	

a No longer used by Medicare.

Many states^37^ and payers^38^ have additional billing restrictions including: (1) requiring patients to sign a telemedicine consent form, (2) receipt of a preauthorization from the insurance company, (3) requiring patients to first have a face‐to‐face encounter with the TMH provider, (4) limitations on provider type, (5) limitations on the type of clinic setting (for originating and/or distant‐sites), and (6) the rurality or shortage area designation of the originating‐site's location. These requirements do not necessarily need to be justified at time of billing, but they could be subject to audit and therefore should be documented in the EHR.

## Supplemental Payments under the Prospective Payment System (PPS)

The cost of contracting with the medical school for a TMH encounter is likely to be substantially higher than the amount that will be reimbursed by Medicaid because of their additional education and research missions. Fortunately, for patients insured by Medicaid, states are required to pay FQHCs their PPS reimbursement rate, which covers 100% of their reasonable costs of providing services. Under the PPS cost reconciliation arrangement, Medicaid makes periodic supplemental payments (also known as wraparound payments) to FQHCs that reflect the difference between reimbursements and their PPS rate.^35^, ^39^ Depending on state Medicaid and/or regional CMS policy, TMH encounters should be eligible for inclusion in cost reconciliation. The “practice address” for the TMH provider must be the FQHC address for the encounter to be eligible for PPS. It is also important to note that many states use an Alternative Payment Methodology, but the supplemental payments in these states must be equal to or exceed the PPS rate.

### Contracting

Contracting for TMH providers may require payment for a minimum number of hours each month even if the monthly volume is not met. Because of other demands on TMH provider time, the medical schools may also require that TMH slots are for a set‐aside time period (eg, 8–11 am on Tuesday mornings). Less flexible appointment scheduling options may result in higher no‐show rates. This is an important issue because, while the FQHC will be charged for the TMH appointment, no‐shows are not eligible for billing or PPS supplemental payments. Therefore, FQHCs will need to explore strategies to prevent no‐shows such as reminders, providing transportation, and placing limits on the number of no‐shows allowed per patient. FQHCs could also mitigate the impact of no‐shows by substituting patients who are present in the clinic during the scheduled TMH encounter and who need mental health services (eg, open access). FQHCs could also choose to “overbook” TMH patients. Contracting will require negotiating an on‐site FQHC suicide protocol that meets the needs of the off‐site TMH providers. Finally, the contract will need to address cross‐coverage for when the TMH provider is on leave and whether the covering provider needs to be credentialed/privileged, given access to the EHR, and paneled with the insurers.

### Indemnity

Due to the lack of relevant legal precedents, malpractice is a concern when developing TMH partnerships. If the medical school specifies the FQHC as an approved “site of practice” for the TMH provider, it will ensure that their practice plan indemnification coverage extends to the TMH clinical work. However, the medical school's malpractice insurance does not cover the FQHC as an entity if named in a lawsuit. Under current policy, the FQHC is not necessarily covered for TMH services by Federal Tort Claims Act (FTCA).^40^ FTCA, which comes at no cost to the FQHC, grants medical malpractice liability protection to the FQHC and its providers. FQHC providers are considered federal employees and the federal government acts as their primary insurer.^41^

For the TMH providers to be eligible for FTCA, 3 difficult‐to‐meet conditions have to be met. First, the TMH provider must be working full‐time (at least 32.5 hours per week), unless practicing in the fields of family practice, general internal medicine, general pediatrics, obstetrics, or gynecology. It is notable that mental health specialists are not listed among the medical specialties deemed to be exceptions. Second, the covered FQHC and the individual TMH provider must have a documented contractual relationship. This contract cannot be with the TMH provider's employer, even if the corporation is eponymous and consists only of the one TMH provider. HRSA's FTCA Health Center Policy Manual specifically states that “compensation that arises from this contract, such as contracted wages, should be paid by the covered entity directly to the individual contract provider. A contract between a covered entity and a provider's corporation does not confer FTCA coverage on the provider.”^40^ Third, the compensation that arises from this contract must be paid by the covered FQHC directly to the individual TMH provider (ie, not to their employer) and the FQHC must issue a 1099 Form to the TMH provider. Unfortunately, few TMH consultants are full‐time providers for the FQHCs, making them ineligible for FTCA coverage.^40^ Likewise, few state medical schools will want the contracts to be between the FQHC and the individual TMH provider. Moreover, the TMH provider would not be covered by the medical school's indemnity plan and would have to purchase individual coverage. Finally, HRSA does not have a published policy guaranteeing FTCA coverage for telehealth of any specialty. Therefore, due to the threat of lawsuits and the lack of previous legal resolutions, FQHCs engaged in TMH will need to purchase supplementary gap indemnity coverage, which covers the FQHC as an entity.

## Recommendations to FQHCs, State Medical Schools, and Policy Makers

Because of the difficulties described above, many rural FQHCs have been unable to establish TMH programs, leaving their patient populations without adequate mental health coverage. The most common approach to TMH requires the patient to become a patient at the distant‐site. However, this approach is suboptimal because: (1) the patient is burdened with having to navigate to another health care system, (2) the TMH providers do not document in the FQHC EHR (compromising safety and quality), and (3) the distant‐site reimbursement from Medicaid is not eligible for supplemental PPS payments. To overcome these problems, FQHCs have to take the following steps: (1) credential and privilege the TMH providers to practice at the FQHCs, (2) obtain EHR site licenses for TMH providers, (3) expand their Scope of Project, (4) contract with the state medical school, (5) empanel the TMH providers with Medicaid and other insurance companies, (6) ensure that TMH encounters are eligible for PPS, and (7) purchase gap insurance to cover the FQHC from malpractice lawsuits. To facilitate taking these 7 steps, we make the following recommendations to FQHCs and medical schools.

### Recommendations for FQHCs

We recommend that FQHCs amend their bylaws to allow for credentialing/privileging by proxy. We also recommend negotiating with EHR vendors for reduced rates for site licenses that reflect the limited time the TMH providers will use the system. FQHCs should consider joining a HRSA‐funded Health Center Controlled Network (eg, Oregon Community Health Information Network) to facilitate TMH providers’ remote access and minimize licensing costs. FQHCs should communicate with HRSA leadership about the benefits of TMH and encourage them to interpret the requirements for Scope of Project expansion to facilitate adding TMH services. FQHCs and their state primary care associations should negotiate with their Medicaid plans to ensure that TMH services are eligible for PPS. FQHCs should also develop effective strategies for reducing no‐show rates in order to minimize lost opportunities for billing.

### Recommendations for State Medical Schools

We recommend contracting with FQHCs to provide TMH services to fulfill their mission of serving all state residents. Contracting with FQHCs will expand the clinical reach of the medical school throughout the state, thereby garnering greater geographic support for their institution. It will also expand educational opportunities for trainees to obtain clinical experience with clinically complex patients from a diverse range of backgrounds who have limited access to services in their community. Finally, it will give state medical schools the opportunity to conduct research that is generalizable to diverse populations, increasing their chances of obtaining federal research funding and improving the external validity of their research findings. We recommend that medical schools consider a point‐to‐point dispersed model that assigns each TMH provider to a small number of FQHCs to minimize each provider's need to learn new EHR systems and to facilitate the development of strong relationships between the TMH provider and the FQHCs’ primary care providers. Options for TMH provider coverage will need to be considered for cases of absences, family leave, or emergencies.

### Recommendations for Policy Makers

In Table 2 we suggest 10 policy changes that will help eliminate or mitigate many of the barriers to TMH described above.

**Table 2:** Recommended Policy Changes

Barrier Addressed	Relevant Organization	Recommendation	Pros	Cons
Credentialing	The Joint Commission	Partner with the Federation of State Board of Medical Examiners, the Association of State and Provincial Psychology Boards, and other relevant licensing boards to develop a national telemedicine credentialing and privileging organization that can confirm the qualifications and clinical skills of telemedicine providers regardless of where they practice. Alternatively, CMS and The Joint Commission should work to streamline the credentialing by proxy process.	This would greatly reduce the costly, time‐consuming, and duplicative efforts of each FQHC having to conduct their own credentialing and privileging.	Would require a change in federal law to allow federal oversight of a process traditionally provided at the state level. Some health care systems may not be willing to accept the indemnity risk associated with a national organization's credentialing and privileging decisions.
Scope of project	HRSA	Revise policies and/or standardize the interpretation of current policies to facilitate the addition of TMH services into the FQHC Scope of Project. Current policy states that all patients must have access to the TMH providers regardless of location.	Removing this requirement may facilitate the expansion of TMH services even if all FQHC clinic sites do not have the capacity to offer TMH.	Creates an inequity for FQHC patients served in clinic sites without TMH.
Scope of project	HRSA	Allow FQHCs to request additional grant funding to support TMH programs when they expand their Scope of Project. Current policy states that the TMH programs must not require additional funding under the Section 330 Public Health Service Act Health Center Program grant.	Removing this requirement will create a more sustainable financial environment because state medical schools do not have the resources to provide services to more uninsured patients.	Requires additional resources
Remote EHR access	EHR Vendors	EHR vendors should develop products that offer site licenses at reduced rates for part‐time telemedicine providers. Alternatively, FQHCs, state primary care associations, and Health Center Controlled Networks should use collective bargaining to negotiate better rates for site licenses.	New products for part‐time providers will make the cost of site licenses less prohibitive and the return on investment more acceptable.	Offering less expensive site licenses for part‐time providers may reduce profit margins for EHR vendors.
Insurance credentialing	CMS	Should not require that TMH providers repeat the credentialing process to be empaneled when they practice in multiple health care organizations. Private insurers should also eliminate this redundant and burdensome requirement.	Reduces duplicate effort and costs.	None
Billing	State Medicaid Administrators	Should allow FQHCs to renegotiate their PPS rate when TMH services are added to the Scope of Project.	Increasing the PPS rate to account for the additional cost of contracting for TMH services creates a more sustainable financial environment.	Requires additional resources
Billing	CMS	Should require that all states and regions make TMH encounters eligible for the PPS rate. Some states (eg, MI) and CMS regional offices (eg, region 5) have determined that if the TMH provider is not physically located at the FQHC, the encounter is not eligible for supplemental PPS payments. Our recommendation is that all states adopt California's policy that if the distant providers are practicing within the “virtual walls” of the FQHC (ie, credentialed and privileged as an FQHC provider), that the encounter be eligible for PPS.^42^	Making TMH encounters eligible for the PPS rate creates a more sustainable financial environment.	Requires additional resources
Billing	CMS	Should allow FQHCs to bill as distant‐sites for Medicare patients.	Removing this restriction will allow TMH providers credentialed and privileged to practice at the FQHC to receive Medicare reimbursements. Otherwise, the FQHC and the medical school must have different arrangements for Medicaid and Medicare patients.	Requires additional resources
Billing	CMS	Should allow facility fees to reflect the indirect costs at the distant‐site, not just the originating‐site. The contracted rates offered by state medical schools reflect both the direct cost of providing patient care and the indirect costs associated with supporting the TMH provider at the distant‐site (eg, office, computer, electricity, heating).	Allowing billing for indirect costs at the distant‐site creates a more sustainable financial environment.	Requires additional resources
Indemnity	HRSA	Should allow TMH providers who are contracting and credentialed and privileged to practice at FQHCs to be covered under FTCA. This could be accomplished by either removing the stipulation that the TMH provider work full‐time at the FQHC or that mental health specialists be added to the list of provider type exceptions. HRSA should also eliminate the stipulation that the compensation that arises from contracted TMH services must be paid by the covered FQHC directly to the individual provider. Finally, HRSA should provide assurance that telehealth services are eligible for FTCA indemnity coverage.	Allowing TMH providers to be covered by FTCA will eliminate the need for FQHCs to purchase supplemental insurance. This will also allow FQHCs to contract with the state medical schools and for providers to be covered under their medical school's indemnity plan.	None

## Conclusions

There is a vast unmet need for mental health services in rural health care professional shortage areas. The geographically inequitable distribution of mental health specialists dictate that it is not feasible to deliver these services face‐to‐face in most cases. TMH represents the only feasible solution to delivering services to the disadvantaged populations served by rural FQHCs. Yet there are a complex and interrelated gauntlet of barriers to offering TMH services in FQHCs. Given the current regulatory and reimbursement environment, this paper offers tangible suggestions for how to develop a sustainable TMH program between FQHCs and state medical schools. Still, there are numerous barriers to developing sustainable TMH programs and current policies need to change in order to facilitate the expansion of TMH. In following our recommended policy changes, CMS and HRSA could help facilitate the adoption of TMH in FQHCs serving our country's most vulnerable and underserved populations.

An important caveat associated with our recommendations is that state TMH policies vary widely and this may limit generalizability for FQHCs and medical schools in some states. The American Telemedicine Association tracks and reports state‐level variation in policies and proposed legislation (available at http://www.americantelemed.org/policy-page/state-policy-resource-center). Likewise, policies and reimbursement models change over time and this may also limit generalizability in the future. In particular, value‐based financing arrangements such as Accountable Care Organizations may drastically alter billing practices. Although, less dependence on paying for encounters and more emphasis on population health is likely to facilitate TMH in general.
